# Wearable sensors detect childhood internalizing disorders during mood induction task

**DOI:** 10.1371/journal.pone.0195598

**Published:** 2018-04-25

**Authors:** Ellen W. McGinnis, Ryan S. McGinnis, Jessica Hruschak, Emily Bilek, Ka Ip, Diana Morlen, Jamie Lawler, Nestor L. Lopez-Duran, Kate Fitzgerald, Katherine L. Rosenblum, Maria Muzik

**Affiliations:** 1 Department of Psychiatry, University of Vermont, Burlington, VT, United States of America; 2 Department of Psychology, University of Michigan, Ann Arbor, MI, United States of America; 3 Department of Electrical and Biomedical Engineering, University of Vermont, Burlington, VT, United States of America; 4 Department of Psychiatry, University of Michigan, Ann Arbor, MI, United States of America; 5 Department of Psychology, East Tennessee State University, Johnson City, TN, United States of America; Brown University, UNITED STATES

## Abstract

There is a significant need to develop objective measures for identifying children under the age of 8 who have anxiety and depression. If left untreated, early internalizing symptoms can lead to adolescent and adult internalizing disorders as well as comorbidity which can yield significant health problems later in life including increased risk for suicide. To this end, we propose the use of an instrumented fear induction task for identifying children with internalizing disorders, and demonstrate its efficacy in a sample of 63 children between the ages of 3 and 7. In so doing, we extract objective measures that capture the full six degree-of-freedom movement of a child using data from a belt-worn inertial measurement unit (IMU) and relate them to behavioral fear codes, parent-reported child symptoms and clinician-rated child internalizing diagnoses. We find that IMU motion data, but not behavioral codes, are associated with parent-reported child symptoms and clinician-reported child internalizing diagnosis in this sample. These results demonstrate that IMU motion data are sensitive to behaviors indicative of child psychopathology. Moreover, the proposed IMU-based approach has increased feasibility of collection and processing compared to behavioral codes, and therefore should be explored further in future studies.

## Introduction

Childhood internalizing disorders (i.e., anxiety, depression) are a highly prevalent and debilitating problem. Up to 2.1% of individuals experience depressive disorders and up to 19.6% experience an anxiety disorder during childhood [1,2]. Importantly, anxiety and depression are chronic conditions that can start as early as the preschool years [3,4] and impair children’s relationships, development, and functioning [5–9]. If left untreated, childhood internalizing disorders are indicators for significant health problems later in life, including substance abuse [10,11], development of later psychopathology [12–14], increased risk for suicide [15], and substantial functional impairment [16,17]. These negative long-term outcomes reveal the high individual and societal burden of internalizing disorders [18], making early identification of those at risk essential. Ideally, targeted preventative efforts can be employed when they have the highest chance of success [19]. However, research employing current diagnostic assessments has demonstrated that they capture the most severely impaired preschoolers, but miss a significant number of children who may go on to develop clinical impairments [20,21]. Clinicians try to improve these assessments by considering reports from children and their parents, but these are also significantly limited. Self-reports of children younger than eight years are unreliable [22–24] and parental report of child problems are often inaccurate [25–27] due to over-reporting when the parent has a diagnosis, and underreporting unobservable symptoms [28] such as thoughts and emotions [27]. Therefore, there is a significant need for objective markers of internalizing symptoms and diagnoses to aid in early identification of those at high-risk by serving as a clinically-relevant tool to complement current diagnostic assessments for early childhood internalizing diagnoses.

To this end, current trends suggest that an observational methodology [29] should be employed as a means for understanding emerging psychopathology [30]. Observational methods used to assess psychopathology are designed to ‘press’ for specific behaviors and affect [31] and have high research and clinical utility [32]. One such approach, known as a “mood induction task,” engages a child in a short laboratory-based activity meant to induce negative or positive emotions. This method has been widely employed to assess markers of psychopathology using a variety of activities including self-statement, music, suggestion, facial expression, game feedback, social feedback, recall, imagery, administrator behavior, empathy, film, threat and public speaking [33,34]. In fear induction tasks, research suggests temporal phases of fear behaviors including 1) response to potential threat, 2) initial response to a known threat (startle response) [35] and 3) response modulation (attempting to influence emotion response once it has been elicited) [36]. Behaviors during these phases have been identified as underlying markers of risk for anxiety disorders [37–39] and thus can serve as targets for objective measurement.

To provide objective markers of psychopathology, researchers utilize a behavioral coding technique on video recordings of these mood induction tasks. This approach requires that at least two researchers watch the video recordings and assign scores based on child verbalizations or facial and body movements associated with specific emotions (e.g., see [40]). For example, behavioral fear and anxiety codes during negative mood induction tasks have been linked to familial risk for internalizing disorders in young children (e.g., see [41]). However, in some child anxiety disorders (e.g., see [42,43]), the association with parent-reported child fear or anxious/depressive symptoms has been inconsistent (e.g., see [44,45]). Behavior coding has been shown to be able to identify valid risk markers, and importantly, to be the most feasible method developed to objectively measure observable child emotions. However, it has significant drawbacks that limit its clinical utility, including extensive training in a standardized coding manual and the hours required to watch and score video recordings of the task, while also consensus scoring a percentage of participants (often one out of five) to ensure reliability [46].

New wearable inertial sensors present the opportunity to track child movement without the need for extensive training or time to watch and score subject videos. Several recent studies have demonstrated that movement during specific emotional contexts is indicative of risk and severity of psychopathology [47–49,37,50,39]. Specifically, accelerometer data from a single wearable sensor were used to inform summary measures of physical activity in normative and clinically affected populations of children and adolescents, including those diagnosed with ADHD [47], Autism Spectrum Disorder [48], and in children with and without intellectual disabilities [49]. While accelerometers alone may provide sufficient information for quantifying physical activity, a more nuanced characterization of participant motion is enabled by combining a tri-axial accelerometer with a tri-axial angular rate gyroscope, to form an inertial measurement unit (IMU). IMUs provide measurement of the full six degree-of-freedom motion of any body segment to which they are attached, and have been used extensively for characterizing human motion in a variety of clinically affected populations (e.g., see [51–55]). For example, in a previous pilot study, we showed that movement measures computed using data from a belt-worn IMU were related to familial risk for internalizing problems in young children [51].

Therefore, our aim is to examine the validity of a wearable sensor for identifying children with anxiety disorders during a mood induction task. Herein, we explore the use of wearable IMUs and behavioral coding to identify objective markers of internalizing symptoms and diagnoses within this task. Specifically, we hypothesize that children with more internalizing symptoms will display tempered motion, but more fear behaviors during the potential threat phase in line with behavioral inhibition [56], and heightened motion and fear behaviors during the startle and response modulation phases (as supported by [57]). In so doing, we compare data between these measurement modalities and compare each modality to child symptoms and internalizing diagnoses to assess their clinical utility in assessment of developmental psychopathology in young children.

## Method

### Participants

Studies had approval from the University of Michigan Institutional Review Board (HUM00091788; HUM00033838). Participants included 63 children (57% female) and their primary caregivers (95.2% mothers). Participants were recruited from either an ongoing observational study (Bonding Between Mothers and Children, PI: Maria Muzik; *n* = 14) or from flyers posted in the community (*n* = 14) and psychiatry clinics (*n* = 35) to obtain a sample with a wide range of symptom presentations. Eligible participants were children between the ages of 3 and 8 who spoke fluent English and whose caregivers were 18 years and older. Exclusion criteria were suspected or diagnosed developmental disorder, having a serious medical condition, or taking medications that affect the central nervous system. The resulting sample of children were aged between 3 and 7 years (M = 5.25 SD = 1.10), was 65% White non-Latinx, and 82.5% lived in two-parent households. Twenty participants (32%) had an annual household income of greater than $100,000.

Diagnostic interviews were conducted for 61 of the children between August 2014 and August 2015, 21 of whom have had an internalizing diagnosis (current (n = 17) or past (n = 4)). Two children could not be reached to obtain clinical diagnosis after participating in the behavior task. Of the 21 with an internalizing diagnosis the primary diagnoses, based on DSM-IV criteria, were as follows: PTSD (n = 5), anxiety or depression-NOS (n = 5), adjustment disorders (n = 4), SAD (n = 4), phobias (n = 3) and depression (n = 1). Six of those children also had a secondary internalizing diagnosis (phobias (n = 2), social anxiety disorder (n = 2), depression (n = 1), and anxiety-NOS (n = 1)). Of the children with an internalizing disorder, four also had an externalizing disorder (ADHD (n = 3) and ODD (n = 1)). One child without an internalizing diagnosis had ADHD.

### Procedure

Child and caregiver were brought into the university-based laboratory and provided written consent to complete a battery of tasks, which were video recorded for later coding. Caregivers completed self- and parent-report questionnaires while children underwent a series of behavioral tasks in an adjacent room. Prior to the start of the behavioral battery, an IMU was secured to the child’s waist. Behavioral tasks were designed to elicit fear responses and positive affect. A diagnostic interview was also conducted with the caregiver to assess for child psychiatric diagnoses. Participants were compensated for their time. Herein, we consider a subset of data from the larger study. We will examine the behavioral codes and IMU metrics used for characterizing participant behavioral response to a single behavioral task designed to elicit fear (the ‘Snake Task’), as well as the diagnostic interview and questionnaires used to assess internalizing symptoms and diagnoses.

The Snake Task has been shown to induce anxiety and fear in young children [58,59]. This task is standardized and all research assistants were trained to carry out the task according to protocol. The total task duration was approximately 2.5 minutes, and task behaviors were conceptually segmented into two phases based on the Research Domain criteria, an NIMH framework for researching mental health: 1) Potential Threat: The child was led into a dimly lit room, unsure of what was inside while administrator gave scripted statements to build anticipation such as “I have something in here to show you” and “Let’s be quiet so it doesn’t wake up”; 2) Acute Threat: The child was startled by the experimenter bringing the fake snake from inside the terrarium to the child’s eye level several inches from their face. The child was then encouraged to touch the snake if they wanted, to insure it was fake.

### Measures

#### Behavioral codes

Behavior codes were adapted from LAB-TAB codes (e.g., see [40,44]). Positive (positivity) and negative valence (fear, anger, and sadness) were coded along two different channels (non-verbal and verbal reactions). Behaviors were categorized in three different intensities (low: e.g. “wringing hands”, moderate: e.g. “slight withdrawal”, and high: e.g. “definite retreat”) within valence and channel type (i.e., “non-verbal fear moderate”). Coders used behavior coding anchors (i.e. to know when to code for different valence intensities. Event-codes were weighted by intensity (x1 for low; x2 for moderate; x3 for high) using the interval duration and summed to create one score per task per valence per channel per phase.

Behaviors were coded by two blind coders and consensus coding was employed for at least every 5^th^ video (at least 20% of all videos) to prevent drift [46]. Training on codes took approximately 20 hours for both coders to become reliable with the trainer and each other. Coding one three-minute video took approximately 20 minutes. Intra-class coefficients (ICC) between coders ranged from .831 to .929, suggesting excellent reliability. In the current analyses, the non-verbal fear code from the Potential Threat and Acute Threat Phases were utilized (one code from each phase) as they most directly relate to the IMU-derived motion measures. Seven videos were not able to be coded due to poor video quality.

#### Inertial measurement units

**Fig 1:**
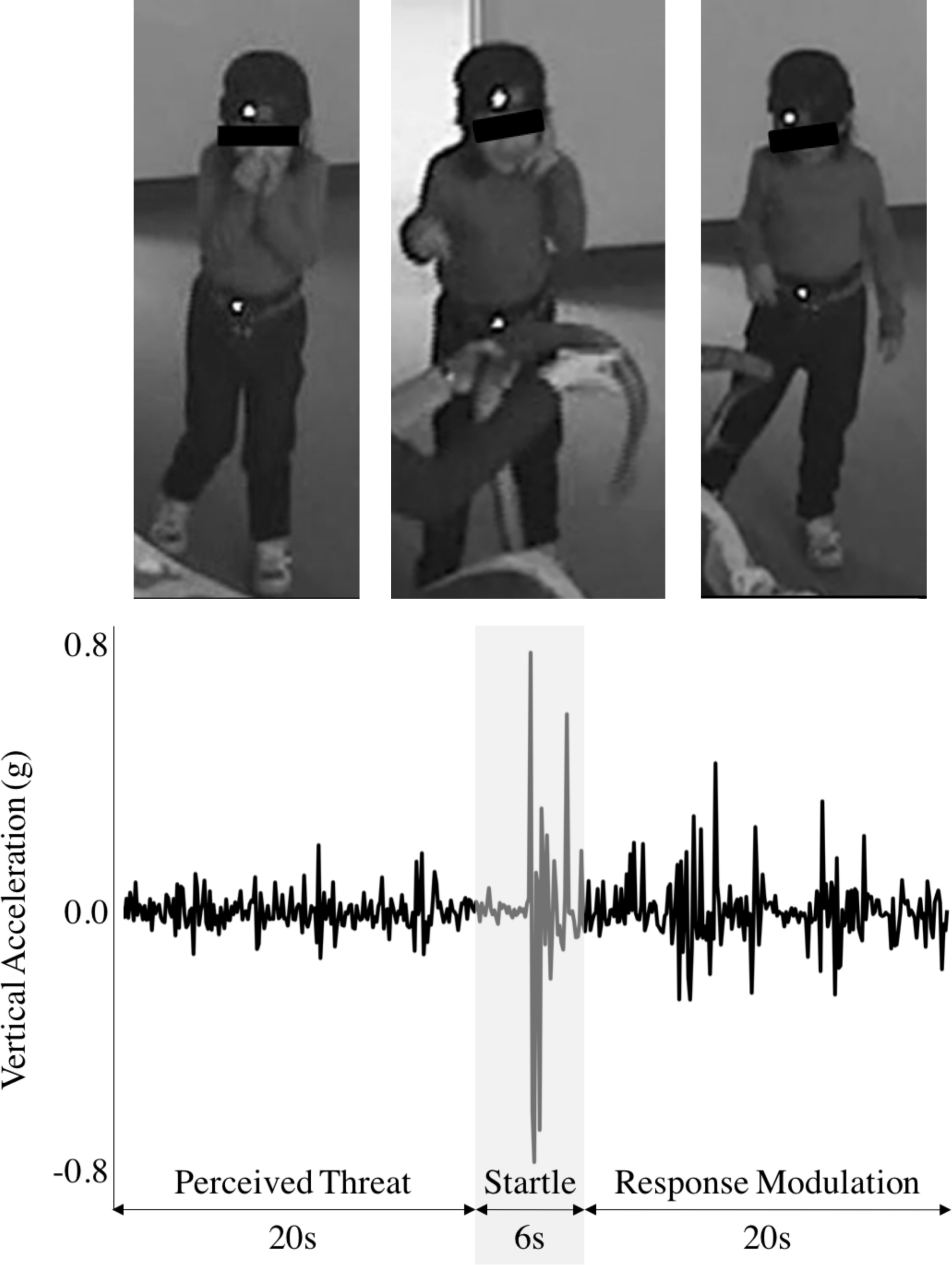
Vertical acceleration measured from waist-worn IMU during mood induction task. Potential threat, startle, and response modulation phases of the snake task (and corresponding images) are indicated for reference. The parent of individual in this figure has given written informed consent (as outlined in PLOS consent form) to publish this photograph.

Participant motion was tracked using a belt-worn IMU (3-Space Sensor, YEI Technology, Portsmouth, OH, USA). Measurements from the accelerometer and angular rate gyro of the IMU were segmented into a Potential Threat phase (20 seconds, from 23 to 3 seconds prior to the moment of startle), and the Acute Threat phase was broken down further into Startle (6 seconds, from 3 seconds prior to 3 seconds post the moment of startle), and Response Modulation (20 seconds, from 3 seconds to 23 seconds post) based on theory [36] (see Fig 1 for a visual representation). Data from each phase were used to compute the 6 summary measures of translational and rotational motion (see [51] for a detailed description of their computation) defined in Table 1, yielding a total of 18 variables to consider for each subject. IMU data processing was performed in MATLAB (Mathworks, Natick, MA, USA), using source code available from [60]. This processing methodology currently requires manual identification of the timing of the startle response in the IMU data. This time was identified by 2 additional blind coders who compared task videos to IMU data. Processing and consensus coding for each video took 2–5 minutes, totaling approximately 5 hours for two research assistants. However, if the startle instant were identified automatically in the data, through a manual trigger mechanism (e.g., see [61]) or the like, this processing time would reduce to 2–5 seconds per subject.

**Table 1:** Definition of the summary measures computed using IMU data from each phase of the mood induction task.

IMU Measure	Description
*ah*_*rms*_	Intensity of subject motion in the horizontal plane
*av*_*rms*_	Intensity of subject motion in the vertical direction
*ωh*_*rms*_	Speed of subject leaning forward, backward, left, and right
*ωv*_*rms*_	Speed of subject turning left and right
*α*_*rom*_	Magnitude of subject leaning forward, backward, left, and right
*γ*_*rom*_	Magnitude of subject turning left and right

Motion measures capture the full six degree-of-freedom motion of each subject in terms of motion intensity, speed, and magnitude during the Potential Threat, Startle, and Response Modulation temporal threat phases. They include root-mean-squared (rms) translational acceleration in the horizontal plane (*ah*_*rms*_) and vertical direction (*av*_*rms*_), rms angular velocity in the horizontal plane (*ωh*_*rms*_) and vertical direction (*ωv*_*rms*_), range of torso tilt motion (rom) relative to the vertical direction (*α*_*rom*_), and range of subject yaw angle (*γ*_*rom*_).

#### Clinical interview

Trained clinical psychology doctoral students, or postdoctoral fellows conducted structured clinical interviews with caregivers. The current study used a version of The Schedule for Affective Disorders and Schizophrenia for School-Age Children (Present and Lifetime Version) modified for use with preschool aged children [62]. In this diagnostic interview, the clinician spent 1–2 hours with the caregiver assessing symptoms of past and current psychiatric disorders.

Interviewers received monthly (or more frequent) supervision by a licensed psychologist and psychiatrist, wherein all cases were reviewed by all clinicians and the supervisor. Final diagnoses were derived via clinical consensus using the best-estimate procedures [63] to integrate a holistic picture based on child and parent report, family history, and other self-report symptom checklists.

#### Questionnaires

The Child Behavior Checklist (CBCL) is a parent-completed questionnaire designed to assess child problem behaviors [64]. The scale consists of 120 items related to behavior problems across multiple domains. Items are scored on a 3-point scale ranging from “not true” to “often true” of the child. Responses result in global T scores for externalizing, internalizing, and total problems, as well as a number of empirically based syndrome scales and disorder-based scales. Only scales available in both versions (ages 1.5–5 and 6–18) were used in subsequent analyses. The CBCL has well established validity and reliability (see [65]).

Subject demographic information was collected using a questionnaire that includes questions regarding child race, gender and family income.

#### Data processing and statistics

Descriptive statistics were computed for all motion variables (IMU and coded), and if skewness was greater than 1 and/or kurtosis was greater than 3, variables were winzorized at approximately 5%. If winzorized variables continued to have high skewness or kurtosis, they were transformed. IMU motion variables were log transformed. See Table 2 for cleaned descriptive statistics of IMU-derived movement variables and behavioral codes. Raw IMU data and behavioral codes included in supporting information S1 File.

**Table 2:** Descriptive statistics of IMU-derived movement variables and behavioral fear codes during mood induction task.

	**Potential Threat**		**Startle**		**Response Modulation**	
**Variables**	**Range**	**Mean (SD)**	**Range**	**Mean (SD)**	**Range**	**Mean (SD)**
*ah*_*rms*_ (m/s^2^)	0.32–2.27	1.02 (0.52)	0.30–4.00	1.52 (0.97)^L^	0.32–3.30	1.17 (0.77)^L^
*av*_*rms*_ (m/s^2^)	0.23–2.41	0.77 (0.59)^L^	0.21–4.00	1.24 (1.01)^L^	0.23–3.00	0.92 (0.74)^L^
*ωh*_*rms*_ (deg/s)	1.29–49.00	19.41 (11.36)^L^	1.80–84.00	29.59 (23.88)	4.39–72.00	24.82 (18.53)^L^
*ωv*_*rms*_ (deg/s)	9.00–59.00	27.07 (12.85)	1.20–85.00	31.81 (19.16)	4.73–61.00	26.24 (14.44)^L^
*α*_*rom*_ (deg)	6.00–59.00	20.47 (14.54)^L^	1.97–32.00	13.36 (8.05)	6.63–62.00	22.43 (14.76)
*γ*_*rom*_ (deg)	16.00–155.00	82.84 (36.87)	1.16–110.00	47.31 (31.06)	7.50–164.00	70.42 (46.32)
	**Potential Threat**		**Acute Threat**			
	**Range**	**Mean (SD)**	**Range**		**Mean (SD)**	
Behavioral Fear	0.00–20.00	9.27 (4.55)	0.00–22.00		8.39 (5.05)	

Descriptive statistics including Range, Mean, and Standard Deviation (SD) for each winzorized IMU-derived movement variable (n = 63) and Behavioral Fear code (n = 56) during each phase of the Snake Task.

^L^ indicates variables log transformed for subsequent analyses.

Paired sample t-tests were employed to assess differences in each measure across phases (Potential Threat, Startle, and Response Modulation for the IMU-derived movement variables, and Potential Threat and Acute Threat for the behavioral fear codes). Pearson product moment correlation coefficients were used to examine the linear relationship between IMU-derived movement variables and behavioral fear codes, and between each measurement modality and child age and gender, as well as parent-reported child symptoms. Independent sample t-tests and linear and logistic regressions are used to investigate the relationships between IMU-derived movement variables, behavioral fear codes, and parent-reported child symptoms and clinician-reported child internalizing diagnoses.

Prior to hypotheses testing, we conducted correlations of IMU motion data and behavioral fear codes with child age and gender. There was a significant Spearman correlation (r = -.275, p = .040) between the behavioral fear code during Potential Threat phase and child gender such that boys were coded as demonstrating less anticipatory anxiety than girls. There were no other significant correlations among IMU motion data or behavioral fear codes with child age and gender.

## Results

### Temporal patterns in IMU motion data and behavioral fear codes

Across the three time phases used to define the IMU-derived movement variables, children tended to significantly increase from Potential Threat to Startle, and decreased again during Response Modulation for acceleration and angular velocity in the vertical direction and horizontal plane (*ah*_*rms*_,*av*_*rms*_,*ωh*_*rms*_,*ωv*_*rms*_). In contrast, tilting and turning range of motion (*α*_*rom*_,*γ*_*rom*_) significantly decreased from Potential Threat to Startle, and increased to baseline during Response Modulation. With the exception of vertical angular velocity (*ωv*_*rms*_, which increased from the first to last phase), motions did not significantly differ between Potential Threat and Response Modulation. For behavioral fear codes, there was no significant difference between Potential Threat and the combined Startle/Response Modulation (Acute Threat) phase. Mean difference between phases, and t-statistic and significance of the difference are indicated in Table 3.

**Table 3:** Changes in IMU-derived movement variables and behavioral fear codes across threat response phases.

	PT—S	S—RM	PT—RM
Variables	*t* (p value)	*t* (p value)	*t* (p value)
*ah*_*rms*_	-4.01 (.001)	3.27 (.002)	-1.43 (ns)
*av*_*rms*_	-3.62 (.000)	2.86 (.006)	-1.38 (ns)
*ωh*_*rms*_	-3.52 (.045)	1.74 (.087)	-2.12 (.038)
*ωv*_*rms*_	-2.05 (.001)	2.49 (.016)	0.40 (ns)
*α*_*rom*_	4.02 (.000)	-5.35 (.000)	-0.84 (ns)
*γ*_*rom*_	7.52 (.000)	-3.76 (.000)	1.81 (.076)
Behavioral Fear	1.15 (ns)		

T-statistics (t) and p values for each IMU-derived movement variable from Potential Threat to Startle (PT–S), Startle to Response Modulation (S–RM), and Potential Threat to Response Modulation (PT–RM) phases of the Snake Task (n = 63). T statistics and p values for Behavioral Fear from Potential Threat to Acute Threat (combined Startle/Response Modulation) phase (n = 56).

*ns indicates p values > .10.

### Associations between IMU motion data and behavioral fear codes

IMU-derived movement variables were unrelated to concurrent behavioral fear codes during the Potential Threat phase (see Table 4). However, all six IMU variables during Startle were positively correlated with behavioral fear codes during the Acute Threat phase. IMU-derived acceleration in the vertical direction and horizontal plane (*av*_*rms*_,*ah*_*rms*_), and angular velocity in the vertical direction (*ωv*_*rms*_, trend level) during the Response Modulation, were also positively correlated with behavioral fear during the Acute Threat phase.

**Table 4:** Pearson correlations between behavioral fear codes and IMU-derived movement variables.

	Potential Threat Behavioral Fear Code *r* (*p* value)	Acute Threat Behavioral Fear Code *r* (*p* value)	
Motions	PT	S	RM
*ah*_*rms*_	0.13 (ns)	0.59 (.000)	0.30 (.024)
*av*_*rms*_	0.10 (ns)	0.52 (.000)	0.30 (.025)
*ωh*_*rms*_	0.07 (ns)	0.46 (.000)	0.20 (ns)
*ωv*_*rms*_	0.10 (ns)	0.53 (.000)	0.26 (.050)
*α*_*rom*_	-0.12 (ns)	0.27 (.047)	0.15 (ns)
*γ*_*rom*_	0.02 (ns)	0.37 (.000)	0.19 (ns)

Pearson product moment correlation coefficients between IMU-derived movement variables during the Potential Threat (PT), Startle (S), and Response Modulation (RM) phases, and their concurrent behavioral fear codes during the Potential Threat and Acute Threat phases (n = 56).

*ns indicates p values > .10.

### Associations between child movement and parent-reported symptoms

All IMU-derived movement variables during the Potential Threat, Startle, and Response Modulation phases were correlated with parent-reported child externalizing symptoms (see Table 5). There were no associations between IMU-derived movement variables and parent-reported internalizing symptoms, except for a trend-level association (*r* = .249, *p* = .053) with turning range of motion (*γ*_*rom*_) during the startle phase. There were no significant correlations between the behavioral fear codes and internalizing or externalizing symptoms.

**Table 5:** Correlations between parent-reported child behavior problems, child movement, and coded behavioral data.

Variables	CBCL Internalizing *r* (*p* value)			CBCL Externalizing *r* (*p* value)		
	PT	S	RM	PT	S	RM
*ah*_*rms*_	0.09 (ns)	0.08 (ns)	0.05 (ns)	0.40 (.001)	0.31 (.014)	0.33 (.009)
*av*_*rms*_	0.12 (ns)	0.12 (ns)	0.12 (ns)	0.40 (.002)	0.38 (.002)	0.32 (.011)
*ωh*_*rms*_	0.08 (ns)	0.16 (ns)	0.12 (ns)	0.49 (.000)	0.50 (.000)	0.36 (.004)
*ωv*_*rms*_	0.20 (ns)	0.12 (ns)	-0.001 (ns)	0.50 (.000)	0.35 (.006)	0.38 (.002)
*α*_*rom*_	0.13 (ns)	0.17 (ns)	0.12 (ns)	0.41 (.001)	0.53 (.000)	0.35 (.006)
*γ*_*rom*_	0.12 (ns)	0.25 (.053)	0.04 (ns)	0.37 (.004)	0.38 (.002)	0.32 (.012)
Behavioral Fear	-0.07 (ns)	0.08 (ns)		0.004 (ns)	0.13 (ns)	

Pearson product moment correlation coefficients between IMU-derived movement variables (n = 61) during the Potential Threat (PT), Startle (S), and Response Modulation (RM) phases, and concurrent behavioral fear codes (n = 54) during the Potential Threat and Acute Threat phases, and CBCL Internalizing and Externalizing symptoms.

*ns indicates p values > .10.

### Associations between child movement and internalizing diagnosis

Five of six IMU-derived movement variables (*γ*_*rom*_ is trend level) were significantly greater for children with clinician-reported history (past or current) of internalizing diagnoses (n = 21) than those without a history of internalizing diagnoses (n = 40, see Table 6). Specifically, during the Potential Threat phase, children with a history of internalizing diagnosis had significantly higher acceleration and angular velocity in the vertical direction and horizontal plane (*ah*_*rms*_,*av*_*rms*_,*ωh*_*rms*_,*ωv*_*rms*_) with medium to large effect sizes. During the Startle phase, bending range of motion (*α*_*rom*_) was significantly greater for children with a history of internalizing diagnosis than those without. Removing children with externalizing disorders (yielding 17 with an internalizing disorder and 39 without) from these analyses did not alter results for the Potential Threat phase. However, bending range of motion during the Startle phase were no longer significantly different and thus should be interpreted with caution. Behavioral fear codes were not significantly different between children with and without histories of internalizing diagnosis in either the Potential Threat or Acute Threat phases.

**Table 6:** Motion and coded behavioral fear differences between children with and without internalizing diagnoses.

	PT	S	RM
Variables	*t* (p value) Effect Size	*t* (p value) Effect Size	*t* (p value) Effect Size
*ah*_*rms*_	-3.15 (.004) 1.08	-0.46 (ns)	-.73 (ns)
*av*_*rms*_	-3.58 (.001) .93	-0.74 (ns)	-1.32 (ns)
*ωh*_*rms*_	-2.59 (.012) .67	-1.14 (ns)	-1.74 (.087) .45
*ωv*_*rms*_	-2.84 (.006) .74	-0.74 (ns)	-1.08 (ns)
*α*_*rom*_	-1.08 (ns)	-2.03 (.047) .53	-1.99 (.051) .52
*γ*_*rom*_	-1.96 (.054) .51	-1.65 (ns)	-0.92 (ns)
Behavioral Fear	-0.10 (ns)	-1.31 (ns)	

T-statistics (t), p values and effect sizes (when significant) for each IMU-derived movement variable during the Potential Threat (PT), Startle (S), and Response Modulation (RM) phases, and behavioral fear codes during the Potential Threat and Acute Threat phases between children with (n = 21) and without (n = 40) internalizing diagnosis.

*ns indicates p values > .10.

## Discussion

In the current analysis of a three-minute fear-induction task for young children, we were able to capture measures of behavioral response using two objective techniques. In the first, we computed 6 distinct variables that captured the full six degree-of-freedom motion of the child. The 6 movement variables are extracted during each of three, theorized, temporal fear phases using data from a single, waist-worn IMU. The second technique relied on extracting human-coded non-verbal fear behaviors during two temporal phases using a popular, event-based coding manual [40,66]. We explored associations between these two measurement modalities, and between each modality and child symptoms and internalizing diagnoses.

We found mixed results when examining associations between movement measurement modalities. Specifically, there were no correlations between modalities during the potential threat phase (See Table 4). This could be due to limited sensitivity or too little movement to be captured by behavioral codes during this phase [51]. It is also possible that IMU and behavior codes were capturing responses differently. For instance, a child exhibiting “freezing behavior” would have a higher behavioral fear score, but a lower IMU score. IMU motion data during the startle and response modulation phases were significantly, positively correlated with behavioral fear codes during the combined startle/response modulation phase (See Table 4). Children exhibited more active movements during these phases, which could be better suited for behavioral coding, and therefore related more closely to the quantitative measures of the behavioral response provided by the IMU. From the startle event onward, the IMU data and behavioral codes similarly and objectively characterized child movement.

When comparing IMU measures to child symptoms, IMU motion data were related to parent-reported externalizing symptoms (see Table 5). This finding is consistent with studies showing that accelerometer-derived activity counts, a summary measure of total movement, are related to child hyper-activity [47,67]. Our results add to the literature by giving a more nuanced characterization of child motion suggesting that large (*α*_*rom*_,*γ*_*rom*_), fast (*ωh*_*rms*_,*ωv*_*rms*_), or high intensity (*av*_*rms*_,*ah*_*rms*_) motions in the context of fear are indicative of child problem behaviors. Externalizing symptoms across this age range include aggressive problems which describe children as “demanding,” “disobedient,” “has a temper,” “fights,” and “attacks others”. It may be that heightened motions detailed by the IMU data indicate a physiological “fight” response profile which maps onto an aggressive temperament. Alternatively, there could be a bias in parent-reporting in which greater movements, potentially indicating poor gross motor skills/behavioral inhibition, are more often seen as outwardly aggressive behaviors. Future investigation on the incremental validity of IMU motion data over and above parent-reports should be conducted. As heightened externalizing symptoms predict long-term problems, their association with nuanced motions in this context of fear suggest wearable sensors could help objectively identify children at risk without potential for bias.

Interestingly, IMU motion data during the potential threat phase, were associated with clinician-diagnosed internalizing disorders (see Table 6). This finding suggests that the way in which children move in response to perceived threat cues are perhaps indicative of a physiological profile of vulnerability. Alternatively, children with internalizing diagnoses, especially PTSD, are more likely to have encountered traumatic events, and may have adapted their responses to potentially threatening situations differently than those who have not. This is consistent with literature demonstrating that children with internalizing diagnoses attend more to threatening cues than those without diagnoses [38,39]. The salience of motion during the potential threat phase (compared to startle or response modulation) may be due to the type of internalizing diagnoses represented in our sample. For instance, internalizing disorders associated with a heightened response to present stimuli would most likely be a Specific Phobia diagnosis. In the current sample, only three children had a primary diagnosis of Specific Phobia; most had PTSD, depression, or an anxious disorder associated with worry and hypervigilance of unknown environments.

As noted above, we observed that IMU motion data were associated with parent-reported externalizing, but not internalizing symptoms, and with internalizing diagnoses. This is somewhat surprising given that both higher externalizing (*r* = .478, *p* < .001) and internalizing (*r* = .416, *p* = .001) symptoms are associated with internalizing diagnoses in this sample. We speculate that this may be explained by a bias in parent-report compared to clinician rating, such that clinicians may be better able to detect contextual triggers, better categorizing child movement in this context as internalizing. Alternatively, association to clinician diagnosis, but not parent-reported symptoms could be due to the measurement windows, such that parent-reported CBCL measured concurrent symptoms (last 6 months), whereas the clinician rated interview included past diagnoses. Additionally, the CBCL internalizing problem scale may not fully capture many of the diagnoses assessed in the clinician interview, like PTSD or specific phobia. However, future studies should investigate the association of IMU motion data and externalizing diagnoses to explore this speculation further. Overall, the association with internalizing diagnoses further establishes the utility of the IMU-based measurement approach for objectively characterizing child impairment.

In the current study, non-verbal behavioral fear codes were unrelated to child symptoms or diagnoses. While some studies show that a combined verbal/non-verbal fear code was moderately associated with internalizing symptoms [43–45], others show a weak or no such relationship with temperamental fear [66] (i.e. *r* = .15, *p* < .05) or anxiety/depressive symptoms [44,68]. We speculate these null results could be due to several different possibilities. First, [45] Buss (2011) suggests that fear behaviors in low threat contexts best indicate risk for internalizing symptoms. Thus, our moderate-to-high threat Snake Task may not be the optimal task for detecting internalizing risk and future work should investigate IMU data and behaviors indicating fear in specifically low threat contexts to better examine their validity. Also, we examined non-verbal behavioral codes to better draw comparisons to the non-verbal nature of the IMU data, however, previous cited works use a combination of verbal and non-verbal fear codes to relate to internalizing symptoms. In post-hoc analyses, we examined combined verbal/non-verbal fear coding associations with internalizing symptoms and diagnoses and there were no additional significant results. Another possibility could be due to variations in behavior coding. While behavior coding is widely used, there are few standardized manuals, and research labs often create their own manuals in order to adapt codes to specific tasks and hypotheses, or to avoid lengthy/expensive coding trainings. The dearth of standardized codes leads to challenges in comparing and/or replicating results across studies. More quantitative, IMU-based measures and open-source software (see [60] for the Matlab scripts employed for this paper) lowers the bar for replication of these results, and extension of these methods to additional behavioral tasks.

IMUs appear to offer an optimal measurement modality to characterize behavioral response to a fear task compared to behavioral coding. In addition to concurrent association with child internalizing disorders, IMU motion data were also more easily processed to separate the three theorized phases of potential threat, startle, and response modulation. Given an alternate fear theory, it would be feasible to change the phase times and reprocess the data within a few moments. In addition to taking significantly longer to collect and check the reliability of the data using coders, re-processing the data after the fact using different parameters is not possible with behavioral fear codes. Moreover, within the potential threat phase, IMU data were more sensitive to differences in child movement than child behavioral fear codes, as demonstrated by IMU data and not behavioral fear data being indicative of internalizing diagnoses in this sample.

### Limitations

This study is not without limitations. First, we investigated the comparison between IMU motion data and behavioral fear codes post-hoc and thus have different phases (Startle and Response Modulation vs the combined Acute Threat phase). Future research using behavioral codes should separate out Startle responses from Response Modulation responses to better compare with IMU motion data. Additionally, future studies should work to establish construct validity of this fear task in this age range. Although previous literature has established the Snake Task and its variants (including the LAB-TAB Spider task) as eliciting stress hormones and fear behaviors, based on our results, we cannot definitively conclude that our coded fear behaviors and IMU data detect fear specifically, as they may be capturing excitement and/or hyperactivity in the current sample. Future research should replicate and investigate our claims in a larger study with additional fear behavior tasks, including low threat tasks. Additionally, a larger sample size would allow examination of internalizing disorders without the presence of comorbid externalizing disorders, and also specific internalizing disorders to explore whether one disorder type yielded different motions than another.

## Conclusion

In the current study, we find preliminary indications of IMU motion data as an objective marker of concurrent child internalizing diagnosis. With further investigation and replication, motion data could act as a low cost, low effort, and highly feasible research tool, especially when compared to the current standard of behavioral coding. With additional exploration and replication, there is opportunity for the supplementary use of wearable sensors in child mental health clinical assessment.

## Supporting information

S1 File Raw IMU and behavior coding data. (CSV)
